# Protective effects of chebulic acid on alveolar epithelial damage induced by urban particulate matter

**DOI:** 10.1186/s12906-017-1870-5

**Published:** 2017-07-19

**Authors:** Kyung-Won Lee, Mi-Hyun Nam, Hee-Ra Lee, Chung-Oui Hong, Kwang-Won Lee

**Affiliations:** 1 Department of Biotechnology, Korea University Graduate School, Seoul, 02841 Republic of Korea; 2 Department of Ophthalmology, University of Colorado School of Medicine, Aurora, CO 80045 USA

**Keywords:** Urban particulate matter, Chebulic acid, Pulmonary alveolus, Inflammation, Alveolar barrier dysfunction

## Abstract

**Background:**

Chebulic acid (CA) isolated from *T. chebula*, which has been reported for treating asthma, as a potent anti-oxidant resources. Exposure to ambient urban particulate matter (UPM) considered as a risk for cardiopulmonary vascular dysfunction. To investigate the protective effect of CA against UPM-mediated collapse of the pulmonary alveolar epithelial (PAE) cell (NCI-H441), barrier integrity parameters, and their elements were evaluated in PAE.

**Methods:**

CA was acquired from the laboratory previous reports. UPM was obtained from the National Institutes of Standards and Technology, and these were collected in St. Louis, MO, over a 24-month period and used as a standard reference. To confirm the protection of PAE barrier integrity, paracellular permeability and the junctional molecules were estimated with determination of transepithelial electrical resistance, Western Blotting, RT-PCR, and fluorescent staining.

**Results:**

UPM aggravated the generation of reactive oxygen species (ROS) in PAE and also decreased mRNA and protein levels of junction molecules and barrier integrity in NCI-H441. However, CA repressed the ROS in PAE, also improved barrier integrity by protecting the junctional parameters in NCI-H411.

**Conclusions:**

These data showed that CA resulted in decreased UPM-induced ROS formation, and the protected the integrity of the tight junctions against UPM exposure to PAE barrier.

**Electronic supplementary material:**

The online version of this article (doi:10.1186/s12906-017-1870-5) contains supplementary material, which is available to authorized users.

## Background

Epidemiologic studies have found a positive correlation between short-term exposure to elevated levels of airborne particulate matter having a mean diameter of less than 2.5 μm [1] and increased morbidity and mortality in humans [2, 3]. The pulmonary effects of urban particulate matter (UPM) exposure are associated not only with increased hospitalization and premature death but also cardiovascular disease [4]. The extracellular side of the alveolar epithelium, which represents the largest surface area of the body in air-breathing animals and human [5], is exposed to UPM, leading to generation of reactive oxygen species (ROS) [6] and inflammatory cytokines [7] in the epithelial cells. These factors can lead to disruption of epithelial barrier function and increase paracellular permeability by destabilizing tight junctions [8]. Disruption of epithelial tight junctions followed by barrier dysfunction plays a critical role in the pathogenesis of a variety of gastrointestinal, hepatic, pulmonary, kidney, and ocular diseases [9]. The disruption of epithelial tight junctions leads to accumulation of fluid and protein in the alveoli, causing asthma [10] and acute respiratory distress syndrome [11].

Induction of cytokines has been reported in in vivo [12] and in vitro response to exposure to a variety of particles [13, 14]. This response is related release by alveolar macrophages of inflammatory cytokines in the alveolar space [7]. To evaluate the inflammatory response after UPM exposure, we determined the expression levels of tumor necrosis factor-α (TNF- α), interleukin-6 (IL-6), and IL-8 in alveolar epithelial cells. The degree of alveolar epithelial cell (AEC) injury and its recovery are pivotal determinants of the toxicity of UPM treatment [15, 16]. However, little is known about protection by natural compounds against alveolar dysfunction induced by UPM. As a candidate, we used chebulic acid (CA) in this study, which was isolated from *Terminalia chebula* [17]. The fruit of this plant is used in Ayurvedic and Chinese folk medicine for a wide range of conditions, including asthma and heart disease [18, 19]. *T. chebular* extract has been reported to neutralize free radicals [20], and CA is one of its main active constituents [17]. Recently, our group reported that CA inhibits advanced glycation end products (AGEs)-induced crosslinking of collagen [21] and prevents collagen accumulation by ERK-phosphorylation-mediated Nrf2 nuclear translocation [22]. Previous studies demonstrated that UPM can induce apoptosis in relevant lung cells [23]. Therefore, we evaluated the effect of UPM on epithelial barrier integrity prior to damage of AECs and the ability of CA to prevent such injury. We estimated that the inflammatory response in AEC induced by oxidative stress caused damage to the tight junctions, leading to alveolar barrier dysfunction. Hence, the objective of this study was to evaluate the protective effect of CA against UPM-induced damage to AECs.

## Methods

### Materials

The medicinal dried ripe fruit of yellow myrobalan, *T. chebula* (Kyungdong Herb Market, Seoul, Korea), was identified by Dr. B. W. Kang (College of Life and Environmental Sciences, Korea University). Voucher specimens were deposited in the Herbarium of the College of Life Sciences and Biotechnology, Korea University, register number H-358. CA was isolated from *T. chebula* Retz. as reported previously [17]. The purified compound was identified as CA using NMR spectra. CA (99.3% purity) was dissolved in dimethyl sulfoxide (DMSO) at a concentration of 10 mM and stored at −20 °C. This stock was further to a working concentration of 10 μM in culture media. A final concentration of no more than 0.1% *v*/v DMSO was used. UPM standard reference material (SRM 1648a) was obtained from the National Institutes of Standards and Technology (Gaithersburg, MD, USA). Particles were collected in St. Louis, MO, over a 24-month period and combined into a single lot to be used as a standard reference. For some experiments, UPM was freshly dispersed in cell culture medium and treated at a concentration of 10–100 μg/mL for 12 h [24, 25].

RPMI 1640 tissue culture medium, fetal bovine serum (FBS), penicillin-streptomycin, and trypsin-EDTA were purchased from Hyclone (Logan, UT), and 3-(4,5-dimethylthiazol-2yl)-2,5-diphenyl tetrazolium bromide (MTT), 2′,7′-dichlorodihydrofluorescein diacetate (DCF-DA), dexamethasone, rotenone (RO) fluorescein isothiocyanate (FITC)-dextran 4000 and phorbol 12-myristate 13-acetate were purchased from Sigma-Aldrich (St. Louis, Missouri, USA). The enhanced chemiluminescence (ECL) detection kit was purchased from Abclon (Seoul, Korea). Antibodies to zonula occludens (ZO-1) (sc-10,804), occludin (sc-5562), and α-tubulin (sc-586) were purchased from Santa Cruz Biotechnology Inc. (CA, USA). The secondary antibodies anti-rabbit IgG and Alexa Fluor 488-conjugated anti-rabbit IgG were purchased from Cell Signaling (Danvers, MA, USA).

### Cell culture

The human pulmonary alveolar epithelial cell line NCI-H441 was obtained from ATCC (Manassas, VA, USA), and cultured in RPMI 1640 medium containing 2.5 g/L dextrose, 2.383 g/L HEPES, 0.11 g/L sodium pyruvate, 10% FBS (*v*/v), 2.2 g/L sodium bicarbonate, 100 units/mL of penicillin and streptomycin. Cells were plated at 2.4 × 10^5^ cells/mL in RPMI 1640 medium. The cells were incubated at 37 °C in a humidified atmosphere containing 5% CO_2_.

### Cell viability

MTT assay was used to determine the viability in order to process the next other experiments at a concentration of within a range that does not affect the cell death. NCI-H441 cells (2.4 × 10^5^/well) were seeded in 24-well plates for 24 h. Cells were treated with 0.1, 1, and 10 μg/mL of UPM in serum-free medium for 24 h and with 10, 50, and 100 μg/mL of UPM for 6 h. Medium was changed to 5 mg/mL MTT reagent in modified RPMI 1640 medium, and the plate was incubated at 37 °C for 3 h. The medium was discarded, and 200 μL/well of DMSO were added to dissolve intracellular insoluble formazan crystals. The optical density at 540 nm was measured using a multiplate spectrometer, and the results were determined as percentages of the control.

### Measurement of ROS

UPM-induced ROS formation was determined by monitoring conversion of DCF-DA to the fluorescent compound dichlorofluorescin (DCF). NCI-H441 cells were cultured at 2.4 × 10^4^ and 1.0 × 10^4^ cells/well in 96-well plates for 24 h with or without CA (0.5, 1, 5, and 10 μM) pretreatment. Then, the cells were incubated with 10 μM DCF-DA in conditioned medium for 30 min, followed by washing twice in warm phosphate-buffered saline (PBS; pH 7.4) and treatment with 10 μg/mL of UPM for 24 h. Then, the conditioned medium containing UPM was replaced with 100 μL of PBS per well. The DCF fluorescence intensity was measured at an excitation wavelength of 485 nm and emission wavelength of 535 nm using a fluorescence spectrophotometer (VICTOR3™ Perkin Elmer, Waltham, MA, USA).

### RNA isolation and quantitative real-time PCR

NCI-H441 (2.5 × 10^5^ cells/mL) cells were cultured in six-well plates and pretreated with CA (5 and 10 μM) or rotenone (5 μM) for 24 h, before being treated with UPM (100 μg/mL) in conditioned medium for 6 h. Total RNA was extracted from cells using RNAiso PLUS (TAKARA Korea Biomedical Co, Seoul, Korea). cDNA was generated using LeGene Premium Express First Strand cDNA Synthesis System (Legene, Sandiego, CA). Real-time PCR was performed with TOPreal™ qPCR 2× PreMIX SYBR green (Enzynomics, Seoul, Korea) and analyzed using an iQ5 Thermal Cycler (Bio-Rad, CA, USA). Primers are listed in Table 1. mRNA expression levels were normalized to that of β-actin. Products were analyzed in 1.0–1.5% agarose gels under UV light.

**Table 1:** Real-time PCR primer sequences

Gene	Oligonucleotide sequence	Size
TNF-α	sense, 5’-CCCAGGGACCTCTCTCTAATCA-3’ antisense, 5’-GCTACAGGCTTGTCACTCGG-3’	80 bp
IL-6	sense, 5’-GGTACATCCTCGACGGCATCT-3’ antisense, 5’-GTGCCTCTTTGCTGCTTTCAC-3’	81 bp
IL-8	sense, 5’-AGAGTGATTGAGAGTGGACC-3’ antisense, 5’-ACTTCTCCACAACCCTCT-3’	118 bp
ZO-1	sense, 5’-GTGTTGTGGATACCTTGT-3’ antisense, 5’-GTGTTGTGGATACCTTGT-3’	92 bp
Occludin	sense, 5’-GAAGCCAAACCTCTGTGAGC-3’ antisense, 5’-GAAGACATCGTCTGGGGTGT-3’	210 bp
β-Actin	sense, 5’-AGCGAGCATCCCCCAAAGTT-3’ antisense, 5’-GGGCACGAAGGCTCATCATT-3’	285 bp

*ZO-1* zonula occludens, *TNF- α* tumor necrosis factor-α, *IL-6 and-8* interleukin-6 and 8

### Western blot analysis

Equal amounts of protein (65 μg) were separated by 7.5% sodium dodecyl sulfate-polyacrylamide gel electrophoresis (SDS-PAGE) and electrophoretically transferred to polyvinylidene fluoride (PVDF) membranes for 12 h at 50 mA using tank transfer device (bio-rad, Hercules, CA, USA). The membranes were blocked with 5% fat-free dry milk in TBST buffer (25 mM Tris-HCl (pH 7.4), 0.1% Tween-20 and 150 mM NaCl) for 1 h at room temperature then incubated with primary antibodies at 4 °C overnight. After washing five times with TBST at 6 min, the membranes were incubated with secondary antibodies conjugated to horseradish peroxidase (HRP) for 1 h at room temperature and washed in TBST five times. Blots were developed using the enhanced chemiluminescence (ECL) reagent (Abclon, Seoul, Korea). Band intensities were normalized to that of α-tubulin and presented as ratios. Band intensities were quantified using the ImageJ software (National Institutes of Health, Bethesda, MD, USA).

### ZO-1 protein staining

To investigate the effect of UPM on a tight junction protein, ZO-1 of lung epithelial cells NCI-H441 cells (2.4 × 10^5^ cells/well) were seeded on six-well culture plates and pretreated with CA (5 and 10 μM) or rotenone (5 μM). After 24 h, cells were treated with 100 μg/mL of UPM for 6 h. Cells were fixed in 3.7% paraformaldehyde in PBS for 20 min and permeabilized with 0.1% Triton X-100 in PBS for 15 min at room temperature. PBS washing was conducted three times in each steps followed by blocking with 1% BSA in PBS for 1 h, and incubation with and anti-ZO-1 primary antibody in 1% BSA overnight at 4 °C. An anti-rabbit Alexa 488-conjugated secondary antibody was added, plates incubated for 2 h at room temperature, and nuclei were stained with 4’,6’-diamidino-2-phenylindole (DAPI, 500 ng/mL) for 5 min. Stained cells were washed with 1% BSA and visualized using a Confocal Laser Scanning Microscope 700 (Carl-Zeiss, Oberkochen, BA, Germany).

### Transepithelial electrical resistance (TEER) measurement

NCI-H441 cells were seeded onto a 12-mm Transwell with a 0.4 μm pore polyester membrane insert (Corning CoStar Corp., Cambridge, MA, USA) at a density of 2.0 × 10^4^ cells/cm^2^. TEER was measured using EVOMX (World Precision Instruments, Sarasota, FL, UK) and expressed as Ohm*cm^2^ after subtracting the resistance of a blank Transwell insert.

### Paracellular permeability assay

Fluorescein isothiocyanate (FITC)-dextran 4000 Da was used to determine epithelial barrier integrity. NCI-H441 cells were seeded in 12-mm transwell inserts in the presence of 1 μM dexamethasone, and fresh medium replaced every second day until formation of differentiated monolayers, and the maximum level of resistance was reached. At least 30 min before the experiments, medium in the basolateral and apical chambers was replaced with 1 mL and 250 μL of fresh medium, respectively, and then 50 μL of FITC-dextran was added to the apical chamber (final fluorescent tracer concentration 1–3 mg/mL). Plates were incubated at 37 °C in a humidified atmosphere containing 5% CO_2_ for 3–4 h [26]. The diffused FITC-dextran in the basolateral chamber was transferred to a black 96-well plate (100 μL/well). The fluorescence intensity was measured at an excitation wavelength of 485 nm and emission wavelength of 535 nm using a fluorescence spectrophotometer (VICTOR3™ Perkin Elmer, Waltham, MA, USA).

### Statistical analysis

Results are expressed as mean values ± standard deviation (*n* = 3). Different letters indicate significant differences at *p* < 0.05 by Tukey’s multiple comparisons test. All statistical analyses were performed using SAS version 9.4 (SAS Institute, Cary, NC, USA).

## Results

### Cell viability

**Fig. 1:**
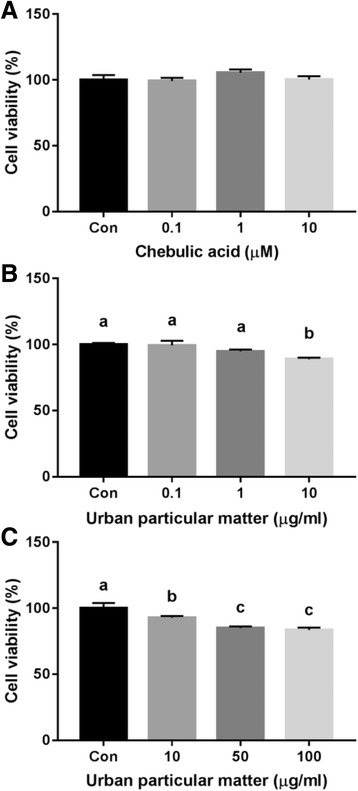
Effect of chebulic acid (CA) and urban particulate matter (UPM; NIST 1648a) on cell viability. Effect of CA on the human pulmonary alveolar epithelial cell (NCI-H441) viability after 24 h (**a**). Cytotoxicity of UPM (0.1, 1, and 10 μg/mL) on NCI-H441 after 24 h (**b**). Effect of exposure to UPM (10, 50, and 100 μg/mL) on NCI-H441 for 6 h (**c**). Data are means ± SD of three experiments with triplicate samples and different letters indicate significant differences at *p* < 0.05 by Tukey’s multiple comparisons test

Compared with the control group, there was no significant difference in NCI-H441viability after treatment with 0.1, 1, and 10 μM CA for 24 h (Fig. 1a). Incubation with UPM (0.1, and 1 μg/mL) for 24 h had no significant effect on cell viability (Fig. 1b). However, UPM (10, 50, and 100 μg/mL) for 6 h showed concentration dependent reduction in cell viability (Fig. 1c). We found that more than 80% of cells were viable with 100 μg/mL of UPM and used this concentration for this study.

### ROS scavenging activity

**Fig. 2:**
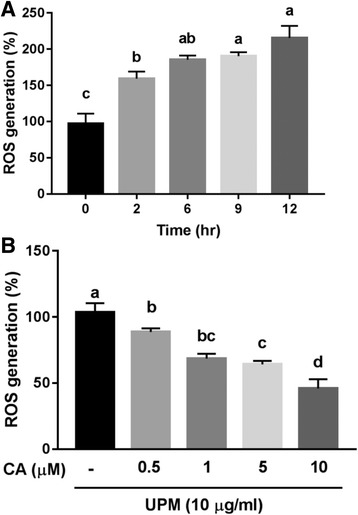
Inhibition by CA of UPM-induced intracellular ROS generation. Effect of exposure to UPM (10 μg/mL) for 0 to 12 h on (**a**) NCI-H441 cells. Effect of exposure to UPM (10 μg/mL) for 12 h after pretreatment with CA (0.5, 1, 5, and 10 μM) for 24 h on NCI-H441 cells (**b**). Data are means ± SD of three experiments with triplicate samples and different letters indicate significant differences at *p* < 0.05 by Tukey’s multiple comparisons test

Intracellular ROS levels in NCI-H441 were measured by monitoring DCF fluorescence intensity. As shown in Fig. 2a, intracellular ROS generation significantly increased compared to the controls after exposure to UPM (10 μg/mL) for 2 h, and treatment with UPM for 12 h resulted in highly increases in fluorescence intensity in NCI-H441. Also, the inhibitor of mitochondrial respiratory chain complex I, rotenone (RO) strongly suppressed ROS generation compare to the specific inhibitor of NADPH oxidase diphenyleneiodonium (DPI) in NCI-H411 cells (Additional file 1: Figure S1). This data indicates UPM mediates ROS generation by which is derived in alveolar epithelium mitochondria electron transfer system. Thus, we used RO as a positive control in our experiments. To evaluate the inhibitory effect of CA on intracellular ROS formation, we pretreated NCI-H441 with CA (0.5–10 μM) for 24 h, followed by incubation with 10 μg/mL UPM for 12 h. Pretreatments with 5 and 10 μg/mL of CA significantly reduced UPM-induced ROS production in NCI-H441 (Fig. 2b).

### Inflammatory cytokine mRNA expression

**Fig. 3:**
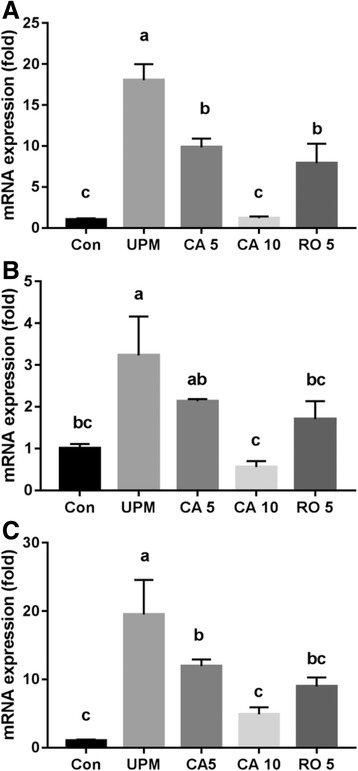
Effect of CA on mRNA expression levels of inflammatory cytokines in the human pulmonary alveolar epithelial cells (NCI-H441) after treatment with UPM. **a** tumor necrosis factor-α (TNF-α), (**b**) interleukin (IL)-6, and (**c**) IL-8 mRNA expression levels of NCI-H441 cells normalized to that of β-actin. After 24 h of pretreatment with CA (5 and 10 μM), NCI-H441 cells were incubated with UPM (100 μg/mL) for 6 h. Data are means ± SD of three experiments with triplicate samples and different letters indicate significant differences at *p* < 0.05 by Tukey’s multiple comparisons test

We determined the effect of CA and RO on the mRNA levels of inflammatory cytokines in NCI-H441. PM induces alveolar type II epithelial cells to release the inflammatory markers TNF-α, IL-6 and, IL-8 [27]. The non-treated group served as a control. A positive control, 5 μM RO was used to inhibit immune responses. After 24 h of pretreatment of CA, cells were incubated with 100 μg/mL of UPM for 6 h. As shown in Fig. 3a, pretreatments with 5 μM CA and RO decreased TNF-α mRNA levels to a similar degree (*p* < 0.05). Moreover, pretreatment with 5 μM CA and 5 μM RO reduced the IL-6 and IL-8 mRNA levels in the presence of UPM (Fig. 3b and c), whereas a higher concentration of CA (10 μM) was required to decrease these inflammatory cytokine mRNA levels.

### Tight junction mRNA and protein expression levels

**Fig. 4:**
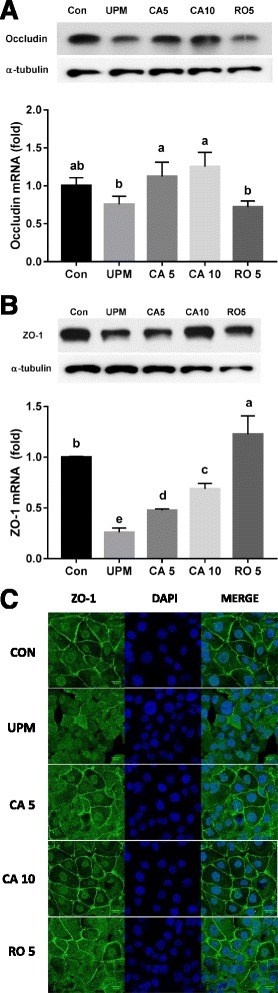
Effect of CA on tight junction expression levels after exposure to UPM. Occludin (**a**) and ZO-1 (**b**) protein and mRNA expression levels in NCI-H441 cells after 24 h of pretreatment with CA (5 and 10 μM) and treatment with UPM (100 μg/mL) for 6 h. **c** Immunofluorescence staining of the effect of UPM (100 μg/mL) and CA (5 and 10 μM) or rotenone (RO, 5 μM) on NCI-H441 cells. After pretreatment with CA (5 and 10 μM) for 24 h, NCI-H441 cells were incubated with UPM (100 μg/mL) for 6 h. Cells were preprocessed and stained with an anti-ZO-1 (1:50) antibody and DAPI (500 ng/mL). ZO-1 is indicated by the white arrow. Data are means ± SD of three experiments with triplicate samples and different letters indicate significant differences at *p* < 0.05 by Tukey’s multiple comparisons test

Tight junctions comprised of peripheral membrane proteins play an crucial role in assembling the transmembrane proteins at the junction sites [28, 29]. ZO-1 and occludin engage roles in the formation of epithelial cell tight junctions [30–33] [33, 34]. Cells were pretreated with 5 and 10 μM CA or 5 μM RO for 24 h and then with 100 μg/mL of UPM for 6 h. As shown in Fig. 4a & b, occludin and ZO-1 mRNA expression levels were lower in the UPM-treated alone group, as determined by real-time RT-PCR. The UPM-induced mRNA levels of these proteins in NCI-H441 cells were recovered by CA in a dose-dependent manner. Especially, the occludin mRNA level was markedly increased in the CA-pretreated group but not the RO-pretreated group. Similarly, the occludin and ZO-1 protein levels in NCI-H441 cells were markedly decreased by UPM treatment, whereas CA or RO pretreatment recovered the levels of these tight junction proteins in cells treated with UPM, and the densitometry analysis of protein levels was shown in Additional file 2: Figure S2. Also, ZO-1-specific immunofluorescence staining was employed to evaluate the formation of tight junctions within NCI-H441 cells. The ZO-1-specific immunofluorescence around cell-to-cell contact sites disappeared following challenge with UPM (100 μg/mL) for 6 h (Fig. 4c). However, pretreatment with CA and RO for 24 h inhibited the UPM-induced disruption of ZO-1.

### Transmembrane epithelial electrical resistance (TEER)

**Fig. 5:**
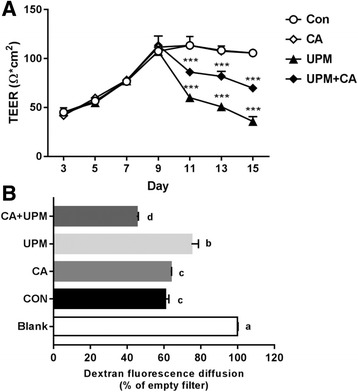
Effect of CA on epithelial barrier integrity after exposure to UPM. **a** Transmembrane epithelial resistance (TEER) was measured using a voltohmmeter. Cells were cultured under non-treated, only CA, only UPM, and CA + UPM treated conditions for 0–15 days. CA (10 μM) was applied on day 8 for 24 h, and UPM (100 μg/mL) on day 9 for 6 h. Asterisks denote significant difference compared to each day control, *p* < 0.001, two-way ANOVA. **b** Paracellular permeability assay was performed using cells cultured with FITC-dextran 4000 Da (final concentration, 0.17 mg/mL). An empty filter was used as the negative control. After pretreatment with 10 μM CA for 24 h, and NCI-H441 cells were incubated with UPM (100 μg/mL) for 6 h. Data are means ± SD of three experiments with triplicate samples and different letters indicate significant differences at *p* < 0.05 by Tukey’s multiple comparisons test

The TEER values of epithelial monolayers of NCI-H441 cells cultured were determined. NCI-H441 cells were seeded onto 12-mm transwell inserts and treated with 1 μM dexamethasone after 24 h. To examine the effect of UPM on the barrier integrity of alveolar epithelial cells, TEER values were measured every second day for 15 days (Fig. 5a). TEER values peaked after 9 days of culture. On day 9, cells were pretreated with 10 μM CA for 24 h, and on the following day, treated with 100 μg/mL of UPM for 6 h. Treatment with UPM alone at day 11 resulted in a TEER value of 60.0 ± 2.8 Ohm*cm^2^. 7However, CA pretreatment resulted in a TEER value 48.5 ± 8.3% higher than that of the UPM only -treated group on day 15.

### Paracellular permeability assay using fluorescence-labeled dextran

Alveolar epithelial monolayer integrity was evaluated by paracellular permeability assay using the aqueous fluorescent tracer, FITC-dextran (molecular weight 4000 Da). The fluorescence intensity of each group was compared to that of the blank (lacking cells) and expressed as a percentage of dextran diffusion (Fig. 5b). CA treatment alone did not alter paracellular permeability compared with the control. However, when the inserts were incubated with UPM for 6 h, the permeability increased by 18.6 ± 4.4% compared with the control. However, the group pretreated with CA followed by UPM treatment exhibited a 36.5 ± 7.9% decrease compared with the group treated with UPM.

## Discussion

The air quality in Seoul (South Korea) on most days is ‘good’ (0–30 μg/m^3^ PM). However, the annual average inhalable PM concentration is 100 μg/m^3^. PM causes chronic airway inflammation by which epithelial cells and alveolar macrophages secreted cytokine and chemokine. Many studies have shown a defense mechanism and response of alveolar macrophage against UPM lead to acute lung injury and chronic obstructive pulmonary disease [35, 36]. Also, many studies have focused on the protective effect of food components against alveolar damage caused by UPM. Because we reported previously that CA isolated from *T. chebula*, which has been used for treating asthma [18], has potent antioxidant activity [17], we hypothesized that CA could protect against the damage to human alveolar epithelial barrier caused by UPM and reduce inflammation in alveolar epithelial cells caused by UPM-induced ROS production. In this study we showed that CA affects epithelial monolayer integrity and paracellular permeability [37]. In addition, the alveolar epithelium participates in immune responses to UPM [38]. So, we focused on disruption of the alveolar epithelial barrier function caused by proinflammatory cytokines and intracellular ROS induced by UPM. RO, which is an inhibitor of electron transfer from ubiquinone binding site to complex I in the mitochondria [39], was used as a positive control in this study and inhibited UPM-induced ROS production, suggesting that intracellular ROS generation by UPM in alveolar cells is mediated by electron transfer in mitochondria complex I. This indicated that damage to the mitochondrial electron transfer system by UPM, especially mitochondria complex I, could be a cellular source of ROS production.

Barrier integrity is critical for maintaining the physiological function of the epithelium in vivo [40]. The alveolar epithelial barrier prevents leakage of intracellular fluids and controls ion transport to promote fluid absorption from the alveolar space [41–43]. The main paracellular permeability characteristics of the epithelium are determined by tight junctions, which are formed by homotypic contacts of the transmembrane protein occludin with ZO-1 [44]. Tight junction proteins are involved in the regulation of diffusion and allow alveolar epithelial cell layers to form selectively permeable cellular barriers that separate the apical and basolateral spaces [44, 45]. In our study, the decreased expression levels of two typical tight junction proteins, ZO-1 and occludin, caused by treatment with 100 μg/mL of UPM were recovered by pretreatment with 5 μM CA. In addition, NCI-H441 cells are capable of forming monolayers of polarized cells and exhibiting a significant TEER [46, 47]. In particular, increased expression of tight junction proteins was related to an increase in the TEER value of epithelial cells from ~45 to ~113 Ohm*cm^2^ and an increase in paracellular permeability to a 4000 Da fluorescent tracer (Fig. 5). Pretreatment with CA prior to exposure to UPM ameliorated the decreased TEER value and reduced efflux of fluorescent tracer from the apical to the basolateral side, compared to treatment with UPM only.

## Conclusions

In this reports, we showed that treatment with CA reduces UPM-induced alveolar epithelium inflammation by suppressing intracellular ROS generation and junctional molecules-related barrier integrity in PAE cell. These results suggested its potential resources of CA for alleviating the damage to the alveolar epithelium caused by PM.

## Additional files

Additional file 1: Figure S1. The effects of specific inhibitors on UPM-induced intracellular ROS generation. Rotenone (RO, 5 μM) and diphenyleneiodonium (DPI, 10 μM) were pre-treated for 1 h, then UPM (10 μg/mL) was treated for 12 h on NCI-H441 cells. RO; mitochondrial electron transport chain inhibitors, DPI; NAD (P) H oxidase inhibitors. Data are means ± SD of three experiments with triplicate samples and different letters indicate significant differences at *p* < 0.001 by Tukey’s multiple comparisons test. (DOC 58 kb)

Additional file 2: Figure S2. Western blot-based quantification of the Occludin (**A**) and ZO-1 (**B**). Western blot analysis (as shown in Fig. 4) was performed to examine the expression of Occludin, ZO-1, and α-tubulin in the cell lysates of NCI-H441 cells. Protein expression quantified by densitometry is shown as relative fold to control normalized to α-tubulin. Data are means ± SD of three experiments with triplicate samples and different letters indicate significant differences at *p* < 0.05 by Tukey’s multiple comparisons test. (DOC 96 kb)

## Abbreviations

CA: Chebulic Acid
DCF-DA: Dichlorodihydrofluorescein diacetate
FITC: Fluorescein isothiocyanate
IL-6: Interleukin-6
IL-8: Interleukin-8
RO: Rotenone
ROS: Reactive oxygen species
SDS-PAGE: Sodium dodecyl sulfate-polyacrylamide gel electrophoresis
TEER: Trans epithelial electric resistance
TNF-α: Tumor necrosis factor-alpha
UPM: Urban particulate matter
ZO-1: Zonula occludins
