# Supplementary figures and images for: Protective effects of chebulic acid on alveolar epithelial damage induced by urban particulate matter
Source: BMC Complement Altern Med. 2017 Jul 19;17:373. doi: 10.1186/s12906-017-1870-5 (PMC5518117; doi:10.1186/s12906-017-1870-5)

**Supplemental Figure S1**

**
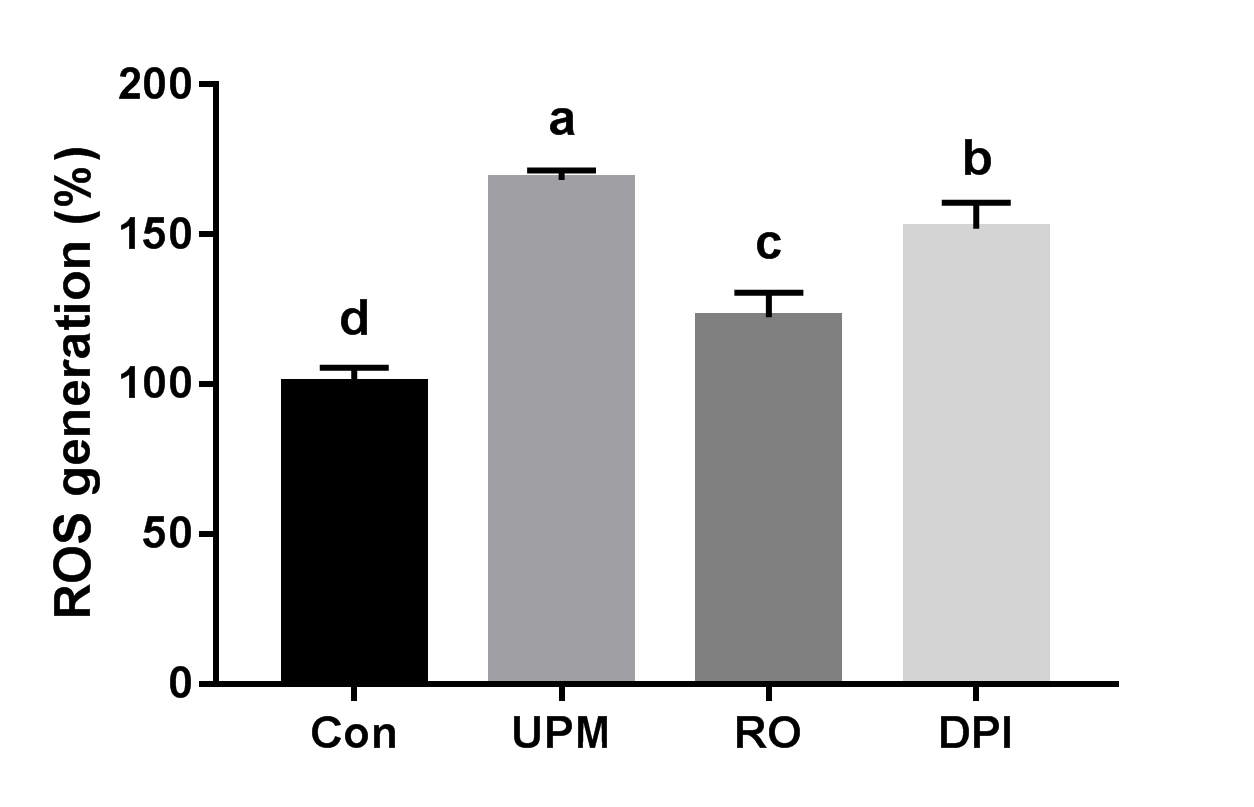
**

Supplement: Supplementary file 1 — The effects of specific inhibitors on UPM-induced intracellular ROS generation. Rotenone (RO, 5 μM) and diphenyleneiodonium (DPI, 10 μM) were pre-treated for 1 h, then UPM (10 μg/mL) was treated for 12 h on NCI-H441 cells. RO; mitochondrial electron transport chain inhibitors, DPI; NAD (P) H oxidase inhibitors. Data are means ± SD of three experiments with triplicate samples and different letters indicate significant differences at p < 0.001 by Tukey’s multiple comparisons test. (DOC 58 kb) [file 12906_2017_1870_MOESM1_ESM.doc]

**Supplemental Figure S2**

**(A)**

**
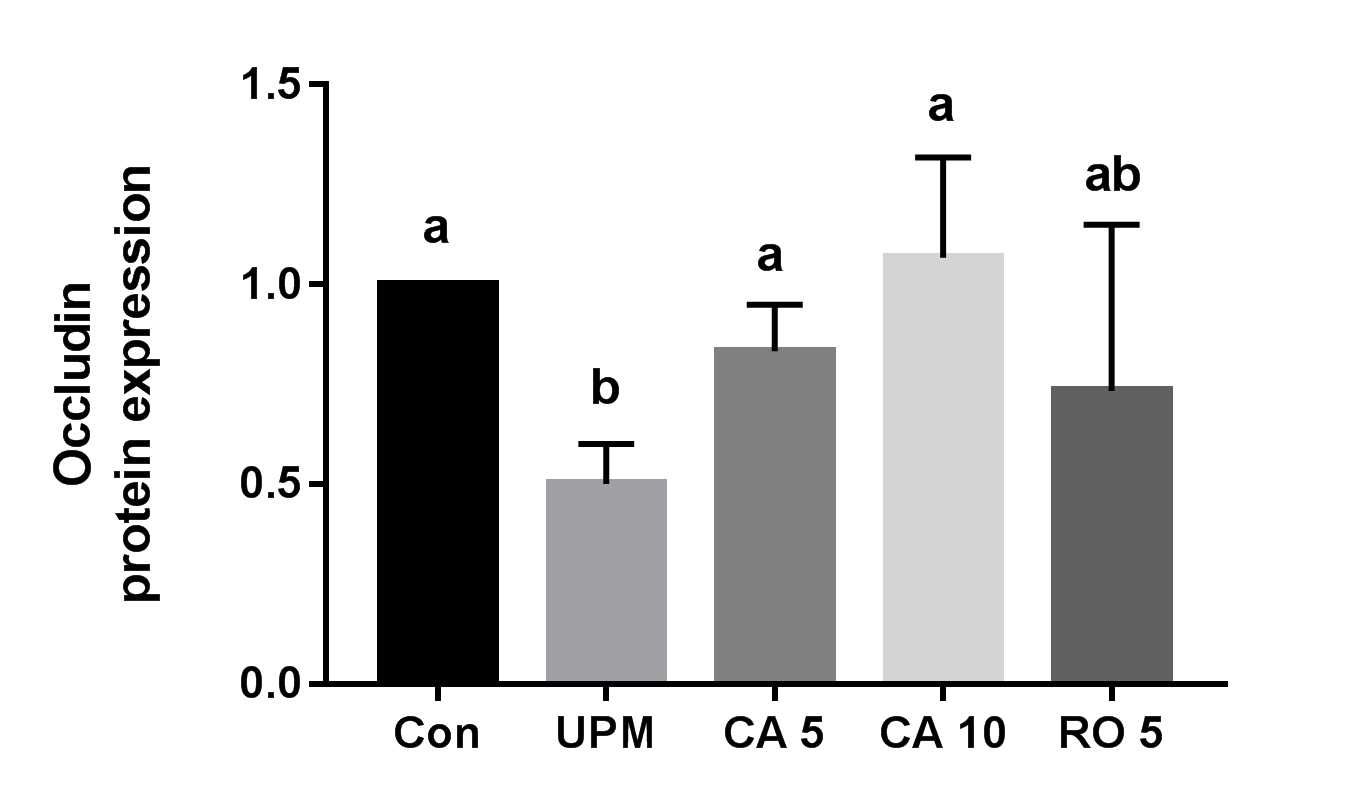
**

**(B)**

**
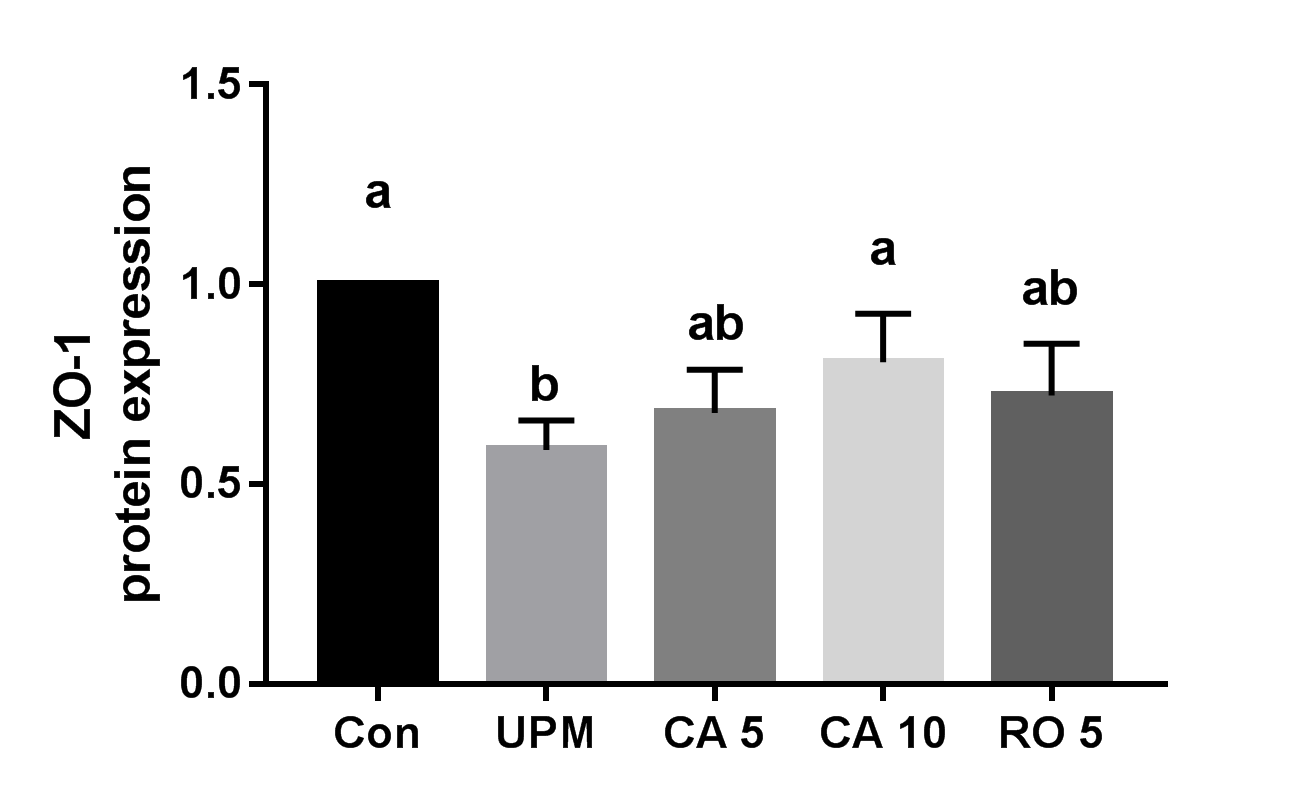
**

Supplement: Supplementary file 2 — Western blot-based quantification of the Occludin (A) and ZO-1 (B). Western blot analysis (as shown in Fig. 4) was performed to examine the expression of Occludin, ZO-1, and α-tubulin in the cell lysates of NCI-H441 cells. Protein expression quantified by densitometry is shown as relative fold to control normalized to α-tubulin. Data are means ± SD of three experiments with triplicate samples and different letters indicate significant differences at p < 0.05 by Tukey’s multiple comparisons test. (DOC 96 kb) [file 12906_2017_1870_MOESM2_ESM.doc]
